# Role of ER Stress in Xenobiotic-Induced Liver Diseases and Hepatotoxicity

**DOI:** 10.1155/2022/4640161

**Published:** 2022-11-04

**Authors:** Yujing Zhang, Yuchen Qi, Shuai Huang, Xiaodong Jiang, Weiwei Xiao, Le Wang, Ziwei Liu, Sulai Liu

**Affiliations:** ^1^Central Laboratory of Hunan Provincial People's Hospital, The First Affiliated Hospital of Hunan Normal University, Changsha, China; ^2^Key Laboratory of Molecular Epidemiology of Hunan Province, School of Medicine, Hunan Normal University, Changsha, China; ^3^Emergency Department, Hunan Provincial People's Hospital, The First Affiliated Hospital of Hunan Normal University, Changsha, Hunan 410005, China; ^4^Department of Hepatobiliary Surgery, Hunan Provincial People's Hospital, The First Affiliated Hospital of Hunan Normal University, Changsha, Hunan 410005, China

## Abstract

The liver is a highly metabolic organ and plays a crucial role in the transportation, storage, and/or detoxication of xenobiotics. Liver damage induced by xenobiotics (e.g., heavy metal, endocrine disrupting chemicals, Chinese herbal medicine, or nanoparticles) has become a pivotal reason for liver diseases, leading to great clinical challenge and much attention for the past decades. Given that endoplasmic reticulum (ER) is the prominent organelle involved in hepatic metabolism, ER dysfunction, namely, ER stress, is clearly observed in various liver diseases. In response to ER stress, a conserved adaptive signaling pathway known as unfolded protein response (UPR) is activated to restore ER homeostasis. However, the prolonged ER stress with UPR eventually leads to the death of hepatocytes, which is a pathogenic event in many hepatic diseases. Therefore, analyzing the perturbation in the activation or inhibition of ER stress and the UPR signaling pathway is likely an effective marker for investigating the molecular mechanisms behind the toxic effects of xenobiotics on the liver. We review the role of ER stress in hepatic diseases and xenobiotic-induced hepatotoxicity, which not only provides a theoretical basis for further understanding the pathogenesis of liver diseases and the mechanisms of hepatotoxicity induced by xenobiotics but also presents a potential target for the prevention and treatment of xenobiotic-related liver diseases.

## 1. Introduction

According to the etiology and pathogenesis, liver diseases include hepatitis virus infections, alcoholic liver disease (ALD), nonalcoholic fatty liver disease (NAFLD) and associated cirrhosis, liver failure (LF), and hepatocellular carcinoma (HCC) [1]. Although great progress has been made in modern medicine regarding prevention, diagnosis, and treatment of liver diseases, these diseases remain a major cause of illness and killing millions of people per year worldwide [2]. Liver diseases in China represent a great proportion of the global burden of liver diseases, with approximately 300 million patients [1], but the specific mechanisms behind almost all liver diseases remain unclear. It is an urgent need to explore the molecular mechanism and etiology of liver diseases for their effective treatment and prevention.

Hepatocytes are enriched in smooth and rough endoplasmic reticulum (ER) in order to perform various metabolic mechanisms, such as plasma protein synthesis and secretion, cholesterol biosynthesis, and xenobiotic metabolism [3], indicating the central role of ER in regulating hepatic metabolism. Thus, the disruption of ER function in hepatocytes, i.e., ER stress initiated by the accumulation of unfolded or misfolded proteins in ER lumen, inevitably participates in the progression of various liver diseases [4]. Both epidemiological and experimental evidences have documented many of the factors relating to hepatic diseases, such as xenobiotics, which are difficult to avoid their global abundance [5–7]. Due to the metabolism of xenobiotics occurring in the liver, hepatocytes have to cope with various perturbations, including ER stress. As a result, an evolutionarily conserved signaling pathway known as the unfolded protein response (UPR) is triggered, which is run by three key factors: (PKR)-like ER kinase (PERK), inositol requiring enzyme 1 (IRE1), and activating transcription factor 6 (ATF6) [8]. The combined activity of all of these processes determines the extent of ER stress and thus causes cells to reestablish homeostasis or activate cell death programs. Hepatocyte apoptosis mediated by ER stress is a pathogenic event in several liver diseases; therefore, the recovery of ER homeostasis before ER stress-mediated hepatocyte apoptosis may provide preventive and therapeutic strategies for xenobiotic-induced hepatotoxicity and hepatic diseases [3].

On the basis of understanding the association between ER stress and cell fate, this review focuses on the role of ER stress in a variety of liver diseases. Additionally, we use the hepatotoxicity of heavy metal environmental endocrine disruptors (EDCs), Chinese herbal medicine, and nanoparticles as examples to discuss the role of ER stress in xenobiotic-induced liver injury. However, our detailed understanding of its mechanism is still not perfect, and the outcome of ER stress cannot be controlled at present. Most of the current research conclusions in this area come from cells and rodent experiments. More exploration is needed to put these conclusions into practice.

## 2. ER Stress and Cell Fate

**Figure 1:**
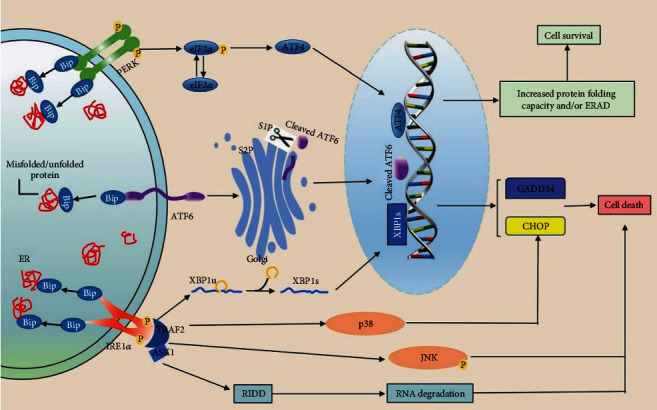
ER stress is related to cell fate in liver diseases. The accumulation of unfolded and/or misfolded proteins could activate UPR. The activation of UPR overcomes disadvantageous effects and restores ER homeostasis via several prosurvival mechanisms, including inhibiting the synthesis of proteins, increasing protein folding or posttranslational modification abilities, and degradation of unfolded or misfolded proteins by the ERAD system. On the contrary, there are some factors or pathway related to ER stress-mediated cell death, including the translation of GADD34 and CHOP, RIDD, and IRE1-activated p38 and JNK signaling.

Protein synthesis, modification, folding, and transportation in the ER are complex regulatory processes. Although the process is tightly regulated, many factors could interfere with the function of this organelle and provoke ER stress with the buildup of unfolded or misfolded proteins. Then, UPR is activated by accumulation signals, attempting to remedy the situation. PERK, IER1, and ATF6 located on the ER membrane coregulate UPR signaling, which determines whether a cell is alive or dead. All three UPR sensors have two domains in the ER lumen and cytoplasm, which bind to ER chaperon glucose-regulated protein 78 (GRP78 or Bip) in ER lumen, maintaining their inactive states. During periods of ER stress, Bip shows a higher affinity to bind with misfolded or unfolded proteins, dissociating from the three sensors. The activation of the UPR overcomes these adverse effects and restores ER homeostasis through several prosurvival mechanisms, including inhibiting the synthesis of proteins, increasing proteins folding or posttranslational modification abilities, and degrading unfolded or misfolded proteins using an ER-associated degradation (ERAD) system [9] (Figure 1). Physiological processes require the UPR to ensure ER homeostasis without triggering cell death pathways. However, above a certain threshold, unresolved ER stress could further lead to cell apoptosis. The mechanisms involved in regulating cell apoptosis under the conditions of irreversible ER stress are now partially understood, and multiple pathways are involved in ER-mediated cell apoptosis [8]. PERK and IRE1 are type-I transmembrane proteins, which could act on their downstream targets after phosphorylation, while type II transmembrane kinase ATF6 needs to be spliced by site 1 protease (S1P) and site 2 protease (S2P) in the Golgi apparatus before translocating into the nucleus to induce the transcription of its downstream target [10]. All three targets can promote apoptosis by activating the transcription of the growth arrest and DNA damage-inducible protein 34 (GADD34) and C/EBP homologous protein (CHOP), which are essential for cell apoptosis. Additionally, a process known as regulated IRE1-dependent decay (RIDD) may unselectively destruct mRNAs at the ER membrane, eventually resulting in cell death due to the depletion of critical enzymatic and structural components of the ER, although this could initially alleviate the protein-folding burden on the ER [9]. Moreover, the phosphorylation of IRE1 can activate the activation of c-Jun N-terminal kinase (JNK) and p38 via apoptotic-signaling kinase-1 (ASK1) by recruiting the tumor necrosis factor associated factor 2 (TRAF2) to induce cell apoptosis [11] (Figure 1).

## 3. Known Effects of ER Stress on Several Liver Diseases

Liver disease is a series of pathological changes at cellular and molecular levels caused by multiple stimulations. There is no effective way to treat hepatic diseases, so it is vital to investigate useful therapeutic targets for such diseases. The central role of the ER in the development of liver diseases continues to emerge in research. Recent studies used rodents and cell models which have demonstrated the connections between liver damage and ER stress as well as UPR [3, 12]. Herein, we listed the function of ER stress in several common liver diseases (Table 1).

### 3.1. ER Stress in Acute Liver Failure (ALF)

ALF is a rare and serious consequence of sudden damage to liver cells and can be fatal within a short period of time. Various damages to liver cells can lead to rapid increase in transaminases, altered mental status, and clotting disorders. The etiology of acute liver failure mainly includes paracetamol (APAP) toxicity, liver ischemia, and viral and autoimmune hepatitis. Hepatotoxicity caused by Chinese herbal medicine is also one of most significant causes of ALF [13]. Animal experiments have shown that APAP-induced ALF is closely related to ER stress-activated JNK1 and JNK2 signaling [14]. Additionally, X-box binding protein 1 (XBP1), a key component of ER stress, is linked to several genes involved in UPR, protein homeostasis, and JNK activation. A study has demonstrated that the activation of PERK-ATF4, IRE1-XBP1, and ATF6 signaling are involved in APAP-induced ALF in mice [15]. A murine ALF model is induced by d-galactosamine (d-GalN, 700 mg/kg)/lipopolysaccharide (LPS, 10 *μ*g/kg), accompanied by the activation of the ER stress-GRP78-CHOP signaling pathway [16], suggesting that the inhibition of CHOP by naphthol is an effective strategy for ALF [16]. These results document that ER stress is associated with the development of ALF, and controlling ER stress-regulated cell apoptosis may be a promising therapeutic strategy. Additionally, another study has demonstrated that the activation of PERK-ATF4, IRE1-X-box-binding protein 1 (XBP1), and ATF6 signaling is involved in APAP-induced ALF in mice [14]. These reports suggest that ER stress plays an important role in the development of ALF.

### 3.2. ER Stress in ALD

ALD attributed to long-term alcohol consumption is one of the major causes of chronic liver disease [17], affecting millions of patients around the world. It has been reported that the metabolite-induced hepatotoxicity of ethanol is the main pathogenesis of ALD. Excessive alcohol consumption promotes hepatic lipid accumulation and hepatic steatosis, accompanied by many pathological changes, such as oxidative stress, ER stress or UPR, and mitochondrial dysfunction [18]. Interestingly, alcohol-induced UPR is sometimes transient and independent of ER stress. Studies performed in alcohol-exposed zebrafish and HepG2 cell model show that the accumulation of misfolded proteins is observed, but most of the downstream targets of UPR are not significantly affected [19]. In addition, Sacks et al. hypothesized that ER does not play primary initiating role in the early stages of ALD, an idea supported by evidence that oxidative stress in early-stage ALD is independent of UPR induction [20].

However, conflicting reports exist regarding the function of ER stress in ALD. The activation of the PERK-ATF4 signaling pathway increased the expression of nicotinamide methyltransferase (NNMT) in response to alcohol exposure in mice models, leading to alcoholic-fatty liver development [21]. In chronic ALD, the PERK-ATF4 signaling pathway induces mitochondrial dysfunction and severe oxidative stress by disrupting the nuclear respiratory factor 1- (NRF1-) mitochondrial transcription factor A (TFAM) pathway in specific gene knockout or overexpression mice with long-term exposure to alcohol [22]. The results from specimens of ALD patients further confirmed the role of ER stress-CHOP signaling in ethanol-induced liver injury [23].

In general, although the effects of ER stress do not play a dominant role in the early stage of ALD, the activated UPR signals increase lipogenesis, oxidative stress, and hepatocyte apoptosis in serious ALD. Inhibiting the activation of ER stress and UPR in this way may be beneficial to the treatment of ALD.

### 3.3. ER Stress in NAFLD

NAFLD is a common liver disease with manifestations of hepatic lipid accumulation, insulin resistance, and inflammation [24]. Despite the research progress that has been made on the pathogenesis of NFALD, its specific molecular mechanism remains obscure. Abnormal lipid accumulation induces the buildup of unfolded and misfolded proteins, activating ER stress, and UPR in hepatocytes [20]. Activated X-box binding protein 1 (XBP1) regulated by IRE1 participates in the progression of NFALD by inducing forkhead box A3 (FOXA3) transcription in vitro and in vivo analysis, directly promoting the expression of lipogenic genes [25]. In addition, the phosphorylation of JNK, which depends on IRE1 signaling, was observed in the liver of obese mice, and the incidence of obesity and insulin resistance significantly decreased in mice with *Jnk1* gene deletion [3]. Similarly, the inhibition of PERK-eukaryotic initiation factor 2*α* (eIF2*α*) by aerobic exercise in mice models with high-fat diet could reduce the development of NAFLD to a certain extent [26]. However, it has been reported that the increased phosphorylation of eIF2*α* is observed in NAFLD, while there is no significant change in the downstream target of PERK signaling [27]. Thus, the association between ER stress and NFALD has been proven through the function of IRE1-XBP1 and eIF2*α* phosphorylation in several reports, but the role of PERK and its target need to be further studied. Notably, there are almost no studies that investigate whether the activated ATF6 is involved in the development of NFALD. Although existing evidence is not enough to fully confirm the effects of UPR on NFALD, it just goes to show that we still need more research to explore the relationship between the two. Based on existing research above, we believe that severity of NAFLD is closely related to the regulation of UPR. UPR may promote the development of NAFLD.

### 3.4. ER Stress in Viral Hepatitis

Viral hepatitis refers to inflammation of the liver caused by infection with a virus. It is caused by the selective infection of the liver through five hepatotropic viruses (HAV, HBV, HCV, HDV, and HEV). In some of these viral infections, acute hepatitis can be cured without intervention, but sometimes, the process progresses to chronic infection [28]. The ER chaperone GRP78 is found to be a key factor that inhibits the replication of HAV in hepatitis A [29]. Activated ER stress, especially the upregulated protein disulfide isomerase (PDI), promotes HBV production by enhancing the use of autophagosome/multivesicular body axis in hepatitis B [30]. On the contrary, the increased ER chaperone tapasin modification is involved in the enhancement of HBV-specific cytotoxic T lymphocyte (CTL) activity, leading to viral clearance in HBV-infected individuals [31]. Moreover, the IRE1-XBP1 signaling pathway plays a significant role in the activation of the HBV S promoter, although it is cell type-restricted [32]. Similarly, ER stress is also involved in cisplatin-induced HBV replication [33]. Notably, current studies on the role of ER stress in HBV are contradictory. For example, another study reported that the activated ERAD system could induce the degradation of the HBV envelop to inhibit HBV replication in HBV-infected HCC cells [34]. The results of methylated RNA immunoprecipitation and sequencing (MeRIP-seq) from HCV-infected samples indicate that viral activation of the ER stress contributes to the changes in m6A *CIRBP*, which is closely related to HCV infection [35]. ER stress can also increase the expression of very low-density lipoprotein receptors, which are one of the HCV receptors, promoting the entry of HCV into hepatocytes [36]. Although there are few studies on the association between ER stress and hepatitis D and E at present, evidence shows that ER stress could produce the mature virion of HDV by inducing the translocation of a large antigen of HDV [37]. Additionally, ER stress participates in the synthesis of a novel viral factor, mediating the efficient replication of genotype-1 HEV [38]. Considering the complex role of ER stress, ER may be a new therapeutic target for the treatment of viral hepatitis.

### 3.5. ER Stress in Liver Fibrosis

Liver fibrosis is the result of repair after repeated liver injury, characterized by the activation of hepatic stellate cells (HSCs) and the deposition of extracellular matrix (ECM) [39]. Synthesizing a large amount of ECM may increase the burden of ER. Surprisingly, UPR is not considered a necessary event in the early stage of HSCs activation, which may be an adaptive response. There is no significant elevation of PERK and IRE1 in the early stage of common bile duct ligation (CBDL) mouse model. Additionally, the idea that ER stress induction is not sufficient to independently activate HSC activation in a model for quiescence further confirms this viewpoint [18]. However, chronic hepatic fibrosis induces the transition of UPR from adaptive response to injury response. An animal study has shown that ATF6 could induce the expression of thioredoxin domain containing 5 TXNDC5, an ER protein PDI, promoting liver fibrosis through the redox-dependent activation of HSCs and excessive ECM production [40]. Moreover, the activation of ER stress in patients with severe liver fibrosis is significantly higher than that in patients with mild liver fibrosis. This may be due to the overexpression of SMAD family member 2 (SMAD2) that is closely related to the development of liver fibrosis. Interestingly, the activation of PERK can induce the expression of SMAD2 via inhibiting its upstream regulator miR-18a [41]. Researchers also found that IRE1-XBP1 signaling promotes the p38-dependent activation of HSCs in liver fibrosis induced by ethanol [42]. Although current studies indicate the regulatory effects of ER stress on liver fibrosis, the role of ER stress in HSCs activation is still intricate and needs to be further investigated.

### 3.6. ER Stress in HCC

Liver malignancy is the fourth leading cause of death in the world. Due to the lack of effective treatment, it continues to cause serious harm to human health [43]. It is well known that ER stress plays different roles in tumors, depending on the tumor type and stage, including HCC. It is found that ER stress markers (GRP78, ATF6, PERK, and IRE1) are upregulated in HCC tissues and negatively correlate with overall survival and clinicopathological scores of HCC patients [44]. In addition, ER stress induces the release of exosomes in HCC cells to increase the expression of programmed cell death ligand 1 in macrophages and inhibit the antitumor function of T lymphocytes, which may be one of the mechanisms for HCC to escape tumor immunity [44]. ER stress cooperating with high-fat diet can also trigger spontaneous, TNF-dependent HCC development [45]. In addition, the secreted protein mesencephalic astrocyte-derived neurotrophic factor (MANF) induced by ER stress inhibits epithelial-mesenchymal transition and NF-*κ*B/Snail signaling pathway to relieve the invasion and migration of HCC, suggesting the conflict function of ER stress in liver cancer [46]. Surprisingly, one study reported that sorafenib could lead to ER stress-induced apoptosis of HCC cells with the DNA release, stimulating the cGAS-STING signaling pathway in CD103^+^ DCs and promoted type I interferon production, thus enhancing the antitumor function of CD8^+^ T and NK cells [47]. Therefore, targeting ER stress to the progression of HCC is theoretically feasible and has great potential, but further research is needed.

## 4. ER Stress and Xenobiotic-Induced Hepatotoxicity

Acute or chronic exposure to xenobiotics may induce hepatotoxicity due to hepatic metabolism [48]. Cells exposed to xenobiotics are in a state of stress, which is a universal mechanism for physiological/pathophysiological significance that often leads to protein-folding defects and altered protein glycosylation, ultimately resulting in ER stress [49]. Therefore, there may be a close association between ER stress and the hepatotoxicity of xenobiotics. Several common xenobiotic hepatotoxicity related to ER stress are listed in Table 2.

### 4.1. Heavy Metal Hepatotoxicity Related to ER Stress

Any kind of xenobiotic detected in the liver is considered very significant since almost all the substances that could be absorbed by the small intestine are first passed on to the liver. Among the hepatotoxicants that have shown key effects on liver function and hepatocyte death, heavy metals are arguably very important. It is well known that the exposure to compounds containing heavy metals causes toxicity in humans and animals. As mentioned, the liver may be the first organ affected by lead (Pb) exposure, either through drinking contaminated water or inhaling Pb emissions. Epidemiological evidence indicates that individuals working in the automobile industry have the highest amount of Pb in their livers, accompanied by elevated ALT and AST from hepatocytes, which are indicators of hepatotoxicity and cell death [50]. In male SD rats, liver injury characterized by increased ALT and AST was detected after Pb acetate was ingested via drinking water at the concentration of 500 mg/L for 8 weeks [51]. Most researchers previously thought that the accumulation of intracellular reactive oxidative stress (ROS) and inflammation was profoundly related to Pb-induced hepatotoxicity, without directly inducing ROS production. However, a recent study reported that Pb appears to cause ER stress-regulated cell apoptosis, strongly indicating that the activated IRE1-mediated JNK signaling pathway is involved in the regulation of Pb-induced hepatocyte apoptosis in rat liver after exposure to an aqueous solution of Pb acetate at a concentration of 500 ppm in drinking water [52]. Additionally, chronic exposure to Pb could disrupt the hepatic and pancreatic glucose metabolism by activating ER stress [53]. Notably, GRP78 from grass carp (*Ctenopharyngodon idella*) provides cytoplasm protection against short-term and low-dose Pb exposure [54].

Among other common heavy metals, chromium (Cr) and its compounds are well-known toxicants that can cause toxicity in various organs, including the liver. As the most toxic form, hexavalent chromium [Cr(VI)] was identified as a class I carcinogen by the International Agency for Research on Cancer in 1990. After a 2011 epidemiological study by Linos et al. showed that long-term exposure to Cr(VI)-contaminated water could increase the incidence of primary liver cancer in local residents [55], researchers began to focus their attention on Cr(VI) hepatotoxicity. There is strong evidence that liver damage could be caused by oral or intraperitoneal injection exposure to Cr(VI) in mice. Our previous study indicated that Cr(VI) exposure could induce acute hepatotoxicity related to liver failure/injury in mice and cell apoptosis [56]. In addition, chronic hepatotoxicity was also the result of low-dose and long-term Cr(VI) exposure, manifested as the premature senescence of hepatocytes [57]. Wang et al. reported the effects of single oral exposure to Cr(VI) in mice liver, showing that Cr(VI) could induce hepatocyte apoptosis in vivo [58]. Another study in rodent models confirmed the chronic hepatotoxicity of Cr(VI). After mice were injected with Cr(VI) for two months, the development of liver fibrosis was found, accompanied by liver structure disorganization, liver dysfunction, and antioxidant enzyme system inhibition [59]. Most present studies argue that the manifestations of Cr(VI) in hepatotoxicity are primarily attributed to oxidative stress, especially ROS accumulation. Recently, evidence emerged that ROS could be a regulator of ER stress and dramatically increases the UPR, and ROS accumulation is often accompanied by ER stress in several diseased conditions [60]. Rats were administered 0.4 mg/kg·bw Cr(VI) five times a week for 30 days, leading to ER stress-regulated hepatocyte apoptosis, characterized by the upregulation of GRP78, CHOP, Cleaved-caspase-12, and ATF6 [61]. Cr(VI) exposure also induced cell apoptosis in hepatocytes via the increase in levels of GRP78, CHOP, and ATF6 in mice [62]. Furthermore, ROS-mediated ER stress closely related to Cr(VI)-induced L02 hepatocyte toxicity via PI3K/AKT signaling pathway [63].

In addition to Pb and Cr(VI), multiple heavy metals also exhibit hepatotoxicity, such as cadmium (Cd) and arsenic (As). Acute and chronic Cd exposure could induce critical liver damage. It has been reported that acute liver damage by Cd exposure is related to hepatic necroinflammation, NAFLD, nonalcoholic steatohepatitis (NASH), fibroplasias, and liver-related mortality [50]. At present, two main pathways provide reasonable evidence for this toxic process, one pathway for the initial injury produced by direct effects of cadmium and the other pathway for the subsequent injury produced by inflammation [64], both of which are closely associated with ER stress. One of the earliest changes in morphology in the case of Cd toxicity is the dilation of ER and ribosome loss [50]. Emerging evidence supports the association between chronic As exposure and abnormal liver function. Both oxidative stress and ER stress are involved in As-induced hepatotoxicity. The ROS-mediated PERK-eIF2*α*-ATF4 pathway is the critical upstream event for subsequent apoptosis induction via CHOP-DR5 signaling in L02 cells with As exposure [65]. Copper (Cu) and nickel (Ni) are essential trace elements that are indispensable in human activity, but excessive exposure to essential trace elements could lead to liver damage. In rodents and cell models, the common hepatotoxic manifestations of these metals are cell apoptosis, which is primarily attributed to oxidative stress, while ER stress apoptosis pathways PERK-ATF4 and IRE1-XBP1 also regulate hepatocyte apoptosis induced by these metals [66].

Altogether, cell apoptosis regulated ER stress, and UPR is profoundly associated with acute and chronic heavy metal hepatotoxicity; therefore, ER may be a target to prevent damages and liver pathologies induced by heavy metals. However, researchers should pay more attention to the specific molecular mechanism of ER stress in heavy metal hepatotoxicity.

### 4.2. ER Stress Involved in EDC Hepatotoxicity

EDCs are a class of xenobiotics defined as “an exogenous chemical, or mixture of chemicals, that can interfere with any aspect of hormone action” [67]. Human health is affected after either individual occupation or dietary and environmental exposure to EDCs. Unlike heavy metals, most of current studies focus on the toxic effects of EDCs on reproduction and development. EDCs perturb the endocrine system, and they are also carcinogenic and hepatotoxic [68], so some researchers explored their hepatotoxicity. Aflatoxin B1 (AFB1), a potent hepatotoxic and carcinogenic agent, is a natural EDC. For oral exposure to 1 mg/kg AFB1 for 4 weeks, the mice experienced liver injury mediated by ROS accumulation [20]. AFB1 also stimulates HCC tumor growth via cell debris from apoptotic cell death regulated by the increased ER stress genes, including *Bip*, *CHOP*, and *PDI* in macrophages; these fragments inhibit macrophage function by eicosanoid and cytokine storm [69]. Synthetic EDCs, such as plasticizer and pesticide, are very common in the environment, and di-(2-ethylhexyl) phthalate (DEHP) is widely used in building materials, food packaging, and medical devices [70]. DEHP in plastics often escapes into the environment as it is not covalently bonded to plastics; the bioaccumulation and environmental persistence of which may result in liver injury. Exposure to DEHP not only induces lipid peroxidation inflammatory cell infiltration in liver depending on the accumulation of ROS but also leads to liver fibrosis regulated by mitochondria-mediated ER stress [71, 72]. Bisphenol A (BPA) is also a widely used plasticizer, which may interfere with the function of the liver. Animal studies indicated a relationship between DEHP-induced hepatic steatosis and chronic ER stress characterized by the upregulation of ER stress-related genes in the liver [73]. The results regarding cells further suggest that BPA could induce the expression of CHOP, Caspase-12, and GRP78 in nonparenchymal hepatocytes, resulting in ER stress-mediated hepatocyte apoptosis [74]. Consumers encounter exposure to pesticide residues by consuming food such as crops, fruits, or vegetables. As the organ of pesticide metabolism, the liver is inevitably damaged in the process. Although studies mainly focus on the role of CYP450, oxidative stress, and DNA damage in pesticide hepatotoxicity, Hutterer et al. investigated the role of ER stress in the hepatotoxicity of pesticides as early as 1968 [75]. Another study on quails indicated the function of activated ER stress in Atrazine-induced hepatotoxicity [76]. In addition, exposure to persistent organic pollutants such as polychlorinated biphenyls (PCBs) could induce liver damage. Recently, a study showed that adolescent exposure to an environmental level of PCBs might induce the development of NAFLD under the regulation of the IRE1-XBP1 pathway in males [77]. In similar, fatty liver results from single exposure to PCBs are associated with increased GRP78 [78]. Although many studies have shown an association between ER stress and EDC hepatotoxicity, the specific molecular mechanism of ER stress in EDC-induced hepatotoxicity is not well-understood.

### 4.3. Effects of ER Stress on Chinese Herbal Medicine Hepatotoxicity

As an essential treatment in traditional Chinese medicine (TCM), Chinese herbal medicine remains prevalent in Chinese medical and healthcare services. As part of the increasing level of research on the use of Chinese herbal medicine in modern medicine, we have found that many bioactive components of Chinese herbal medicine can be involved in the regulation of critical cellular activities, such as oxidative stress, ER stress, and cell apoptosis [79]. Chinese herbal medicine is widely used to treat various ailments, including colds, bruises, and liver diseases. It is well known that Chinese herbal medicine encompasses compounds rich in antioxidants that attenuate oxidative stress, nitroative stress, and ER stress by improving protein folding and cell survival. For example, Hugan Qingzhi Tablet (HQT) and *Schisandra chinensis* (Turcz.) Baill. (SC) extracts have significant protective effects against NAFLD by alleviating ER stress [80, 81]. When Pien Tze Huang and Yinchenhao decoctions were used to treat liver injury induced by alcohol or obstructive jaundice, the inhibition of PERK signaling pathway played a key role [82, 83]. Procyanidin B2 (PCB2), the active ingredient of herbal cinnamon, can also be regarded as a liver protective regent in NAFLD, which could reduce the activation of IRE1 signaling [84]. In addition, the upregulation of the IRE1-JNK pathway is closely related to the therapeutic effect of Bufalin in HCC [85]. Although Chinese herbal medicine has great potential in treating liver diseases, this medicine could inevitably burden or even damage the liver. Emerging evidence shows the hepatotoxicity of Fructus Psoraleae (FP), a traditional herbal medicine that is widely used to treat various skin diseases. In vitro models of flavonoids in FP induced hepatocyte apoptosis via mitochondrion-mediated ER stress [86]. Furthermore, the improper administration of the traditional Chinese medicine cassia seed can cause ER stress-regulated hepatocyte apoptosis because of emodin, an anthraquinone active substance in cassia seed. Emodin can induce the accumulation of ROS, which further leads to intracellular calcium imbalance and ER stress [87]. Altogether, it is necessary to study the therapeutic mechanism and hepatotoxicity related to ER stress for the safe use of Chinese herbal medicine.

### 4.4. Role of ER Stress in Nanoparticle Hepatotoxicity

Human exposure to nanoparticles is inevitable due to their widespread abundance, resulting in nanotoxicology research which is now gaining popularity [88]. It has been reported that exposure to copper oxide nanoparticles (nano-CuO) could lead to liver injury, depending on the activation of CHOP, JNK, and Caspase-12 apoptosis pathways regulated by oxidative-stress-triggered ER stress [89]. Similarly, ZnO nanoparticles (nano-ZnO) show adverse effects on the liver in murine via the increase in phosphorylation levels of PERK and eIF2*α* and the expression of ER stress-related apoptotic proteins to induce hepatocyte apoptosis [90, 91]. Ultrasmall superparamagnetic iron oxide nanoparticles (USPIo-NPS), widely used in the diagnosis of liver disease, also induces liver inflammation by activating the PERK-ATF4 signaling pathway via ER calcium exhaustion [92]. Nanoparticle exposure also induces liver damage in invertebrate. For example, water environmental pollutant copper nanoparticles (CuNPs) could induce fatty liver formation in aquatic organisms via the ER stress-mediated sterol regulatory element-binding protein-1c (SREBP-1c) pathway [93]. Of course, not all nanoparticles are harmful to the liver. Squalene from virgin olive oil is loaded onto PLGA nanoparticles and can accelerate its absorption by cells, exerting protective effects on hepatocytes during oxidative stress and ER stress [94].

## 5. Summary and Future Directions

In general, the understanding of xenobiotic-related liver disease and hepatotoxicity is gradually deepening, but there are still many unknown toxic mechanisms to be explored. ER stress is involved in regulating the fate of cells and is an important participant in the development of xenobiotic-induced liver disease and hepatotoxicity, making it an attractive target for the prevention of some xenobiotic-induced hepatotoxicity. Given that there are many kinds of xenobiotics that are unavoidable in our daily life, further exploration is required.

## Figures and Tables

**Table 1:** The roles of ER stress in liver diseases.

Liver disease	Roles of ER stress	References
ALF	Participates in the development of ALF via JNK1, JNK2, and CHOP	[14, 16]
ALD	Plays a primary role in the progress rather than the early stage of the disease	[19–23]
NAFLD	Promotes the development of NAFLD	[25, 26]
Hepatitis A	Inhibits the replication of HAV	[29]
Hepatitis B	Promotes the production and replication of HBV, inhibits immune response, and inhibits the replication of HBV in HCC cells	[30, 32–34]
Hepatitis C	Contributes to HCV infection	[36]
Hepatitis D	Induces the transfer of HDV large antigen from nucleus to cytoplasm and HDV mutation	[37]
Hepatitis E	Promotes the replication of gene-1 HEV	[38]
Liver fibrosis	Promotes the development of liver fibrosis	[40, 41]
HCC	Dual effect of promoting or inhibiting HCC	[44–46]

**Table 2:** The roles of ER stress in xenobiotic-induced hepatotoxicity.

Type of xenobiotics	Roles of ER stress	References
*Heavy metals*		
Pb	Participates in Pb-induced hepatocyte apoptosis	[52]
Cr	Participates in Cr-induced hepatocyte apoptosis	[61, 62]
Cd	Participates in Cd-induced hepatocyte apoptosis	[50, 64]
As	Participates in As-induced hepatocyte apoptosis	[65]
Cu	Participates in Cu-induced hepatocyte apoptosis	[66]
Ni	Participates in Ni-induced hepatocyte apoptosis	[66]
*EDCs*		
AFB1	Relates to AFB1 hepatotoxicity	[69]
DEHP	Participates in DEHP-induced liver fibrosis	[72]
BPA	Participates in BPA-induced hepatic steatosis and hepatocyte apoptosis	[73, 74]
Pesticide	Sensitive indicator of pesticide hepatotoxicity	[75, 76]
PCBs	Participate in the development of NAFLD and fatty liver caused by PCBs	[77, 78]
Chinese herbal medicine	Therapeutic targets for liver disease and contributes to liver injury of Chinese herbs	[80–87]
Nanoparticles	Closely relate to nanoparticles hepatotoxicity	[89–93]
